# Organic–Inorganic Hybrid Synaptic Transistors: Methyl-Silsesquioxanes-Based Electric Double Layer for Enhanced Synaptic Functionality and CMOS Compatibility

**DOI:** 10.3390/biomimetics9030157

**Published:** 2024-03-03

**Authors:** Tae-Gyu Hwang, Hamin Park, Won-Ju Cho

**Affiliations:** 1 Department of Electronic Materials Engineering, Kwangwoon University, Gwangun-ro 20, Nowon-gu, Seoul 01897, Republic of Korea; wqde748@naver.com; 2 Department of Electronic Engineering, Kwangwoon University, Gwangun-ro 20, Nowon-gu, Seoul 01897, Republic of Korea; parkhamin@kw.ac.kr

**Keywords:** organic–inorganic synaptic transistor, electrical double layer (EDL), neuromorphic computing, thermal/chemical stability, synaptic functionality, CMOS compatibility

## Abstract

Electrical double-layer (EDL) synaptic transistors based on organic materials exhibit low thermal and chemical stability and are thus incompatible with complementary metal oxide semiconductor (CMOS) processes involving high-temperature operations. This paper proposes organic–inorganic hybrid synaptic transistors using methyl silsesquioxane (MSQ) as the electrolyte. MSQ, derived from the combination of inorganic silsesquioxanes and the organic methyl (−CH_3_) group, exhibits exceptional thermal and chemical stability, thus ensuring compatibility with CMOS processes. We fabricated Al/MSQ electrolyte/Pt capacitors, exhibiting a substantial capacitance of 1.89 µF/cm^2^ at 10 Hz. MSQ-based EDL synaptic transistors demonstrated various synaptic behaviors, such as excitatory post-synaptic current, paired-pulse facilitation, signal pass filtering, and spike-number-dependent plasticity. Additionally, we validated synaptic functions such as information storage and synapse weight adjustment, simulating brain synaptic operations through potentiation and depression. Notably, these synaptic operations demonstrated stability over five continuous operation cycles. Lastly, we trained a multi-layer artificial deep neural network (DNN) using a handwritten Modified National Institute of Standards and Technology image dataset. The DNN achieved an impressive recognition rate of 92.28%. The prepared MSQ-based EDL synaptic transistors, with excellent thermal/chemical stability, synaptic functionality, and compatibility with CMOS processes, harbor tremendous potential as materials for next-generation artificial synapse components.

## 1. Introduction

Recent advancements in artificial neural network technologies, particularly in deep learning, have spurred a growing demand for enhanced information processing capabilities. However, the conventional von Neumann architecture, characterized by physically separated CPU and memory components, encounters challenges such as significant energy consumption and slow information processing [1]. These aspects are especially problematic when handling extensive data in artificial intelligence and deep learning, with traditional architectures being unable to perform parallel and distributed processing, resulting in bottleneck issues [2]. To address these challenges, a paradigm shift is underway to introduce novel computing systems capable of overcoming the constraints of traditional architectures.

The human brain, with ~10^15^ synapses and ~10^11^ neurons, exhibits remarkable parallel processing and information storage capabilities with low energy consumption [3,4]. The efficient and highly accurate information processing capabilities of the human brain have fueled significant interest in developing artificial synaptic neuromorphic computing systems that aim to mimic brain functions. Analog electronic devices resembling synapses, capable of emulating the learning and memory functions of the human brain, are essential for the successful implementation of neuromorphic systems. In this context, researchers have developed two-terminal synaptic devices with ferroelectric [5,6,7], memristor [8,9,10], and phase-change [11,12,13] principles for realizing artificial synaptic functionalities. Moreover, three-terminal devices based on the field-effect transistor structure have been introduced. Notably, three-terminal electric-double-layer (EDL)-based synaptic transistor structures, formed at the interface between the gate electrode, electrolyte, and channel, have gained prominence as artificial synaptic devices [14,15]. The migration of internal ions within the EDL electrolyte, in response to the bias applied to the gate electrode, contributes to the modulation of channel conductivity, similar to the functionality of synaptic weights. Owing to these features, EDL-based synaptic transistors effectively simulate synaptic dynamic functions with low power consumption, rendering them promising candidates for artificial synaptic devices. Furthermore, spurred by the growing interest in environmentally sustainable and biocompatible electronic components, research is underway on EDL-based synaptic transistors employing organic materials like chitosan [16,17,18], starch [19,20,21], albumin [22,23,24], pectin [25,26,27], and casein [28] as electrolytes. These organic substances boast abundant natural resources, biocompatibility, and cost-effective solution processing. However, these materials also present a significant drawback: inadequate thermal and chemical stability. This limitation poses challenges for their integration into complementary metal oxide semiconductor (CMOS) processes, known for their high-temperature operations [29].

This study was aimed at addressing these gaps by fabricating EDL-based synaptic transistors using methyl silsesquioxane (MSQ), a polymer organic–inorganic hybrid material, as the electrolyte. MSQ is a widely used organic–inorganic hybrid compound in CMOS processes, featuring an organic methyl group (−CH_3_) bonded to the inorganic silsesquioxane structure. Applying MSQ as the electrolyte in the EDL synaptic transistor helps enhance thermal and chemical stability, overcoming the stability issues associated with conventional organic-based EDL synaptic transistors. Additionally, this framework is compatible with CMOS processes. We verified the EDL properties associated with internal protons in MSQ through frequency-dependent capacitance measurements. Subsequently, we fabricated synaptic devices with indium–gallium–zinc oxide (IGZO), MSQ, and p-Si as the post-synaptic, neurotransmitter, and pre-synaptic components, respectively, and evaluated the corresponding transfer and output characteristics. Additionally, we observed the variation in hysteresis with the increase in maximum gate voltage to confirm the migration of mobile protons in MSQ. The device’s performance in exhibiting synaptic-plasticity-related functions, such as excitatory post-synaptic current (EPSC), paired-pulse facilitation (PPF), signal pass filtering, spike-number-dependent plasticity, and potentiation/depression, were evaluated. Finally, to demonstrate the artificial synaptic neuromorphic computing system, we simulated the training of a handwritten Modified National Institute of Standards and Technology (MNIST) image dataset using an artificial deep neural network (DNN).

## 2. Materials and Methods

### 2.1. Materials

The materials used in this study included p-type Si wafer (resistivity range between 1–10 Ω·cm, LG SILTRON Inc., Gumi, Republic of Korea), 400F spin-on glass (FILMTRONICS Inc., Butler, PA, USA), an IGZO sputter target (In_2_O_3_:Ga_2_O_3_:ZnO = 4:2:4.1 mol%, THIFINE Co., Ltd., Incheon, Republic of Korea), and an ITO sputter target (In_2_O_3_:SnO_2_ = 9:1 mol%, THIFINE Co., Ltd., Incheon, Republic of Korea). Additionally, Al pellets (purity > 99.999%; THIFINE Corp., Incheon, Republic of Korea) were used.

### 2.2. Fabrication of Organic–Inorganic Hybrid MSQ-Based EDL Synaptic Transistors

**Figure 1:**
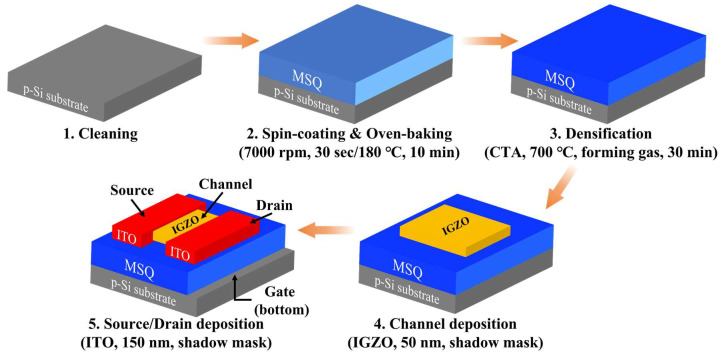
Fabrication flow diagram of organic–inorganic hybrid MSQ-based EDL synaptic transistors.

Figure 1 illustrates the fabrication of the organic–inorganic hybrid MSQ-based EDL synaptic transistor. A 1 cm^2^ × 1 cm^2^ (100)-oriented p-type Si wafer was cleaned following the protocol specified by the standard Radio Corporation of America. First, the organic–inorganic hybrid MSQ was spin-coated onto the cleaned p-type Si substrate at 7000 rpm for 30 s, followed by oven-baking at 180 °C for 10 min to remove the solvent. Subsequently, conventional thermal annealing was performed at 700 °C for 30 min in a forming gas environment to achieve surface hardening and densification of the MSQ film. Next, the IGZO channel layer, with a thickness of 50 nm, was deposited using radio-frequency (RF) magnetron sputtering, employing a shadow mask for patterning. Finally, the source/drain electrodes, composed of a 150-nm-thick ITO layer, were deposited using RF magnetron sputtering with a shadow mask. The working pressure, RF power, and Ar flow for the ITO sputtering process were set as 3 mTorr, 100 W, and 20 sccm, respectively.

### 2.3. Characterization

The frequency-dependent capacitance characteristics of Al/MSQ electrolyte/Pt structure capacitors were analyzed using the Agilent 4284A precision LCR meter (Hewlett-Packard Corp., Palo Alto, CA, USA). The electrical and synaptic properties of the fabricated MSQ-based EDL transistors were evaluated using the Agilent 4156B precision semiconductor parameter analyzer (Hewlett-Packard Corp., USA). Furthermore, the synaptic operation of the fabricated devices was verified by applying synaptic stimuli pulses using the Agilent 8110A pulse generator (Hewlett-Packard Corp., USA). To ensure accurate evaluations, all electrical performance assessments were conducted in a dark box within a probe station to minimize the influence of external light and electrical noise.

## 3. Results and Discussion

### 3.1. Verification of MSQ Electrolyte for EDL Operation

**Figure 2:**
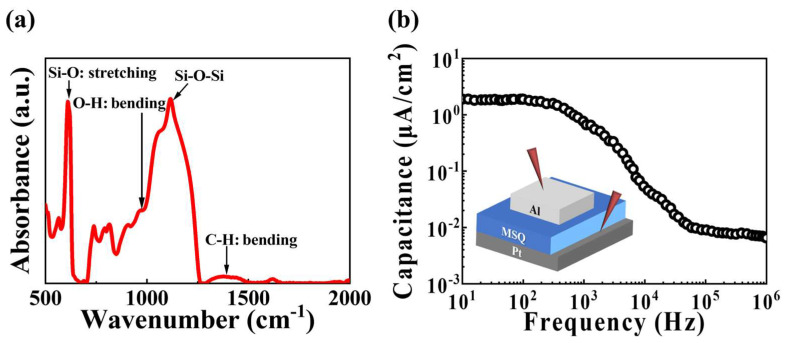
(**a**) FT-IR spectra of the organic–inorganic hybrid MSQ-based EDL synaptic transistor. (**b**) Frequency-dependent capacitance curve of Al/MSQ/Pt capacitors.

Following the spin-coating and thermal annealing of the MSQ film, the presence of specific compounds within the film, as an EDL material, was verified and validated through Fourier transform infrared spectroscopy (FT-IR) analysis. Figure 2a depicts the FT-IR spectrum of the MSQ film in the range of 500 to 2000 cm^−1^, composed of distinct peaks corresponding to specific compounds within the film. The peak at approximately 610 cm^−1^ is attributed to Si–O stretching [30]. Additionally, the O–H bending peak appears at ~960 cm^−1^, contributing to proton conductivity [31]. The peak representing the cage structure of the inorganic components Si and O emerges around 1100 cm^−1^, corresponding to the Si–O–Si structure [32]. Broad peaks in the range of 1300 to 1500 cm^−1^ correspond to C–H bending, originating from the organic methyl group (−CH_3_) of MSQ [31]. These results indicate that the organic–inorganic hybrid MSQ induced ion conduction through mobile protons, thereby verifying the EDL characteristics at the interface [33]. Moreover, Figure 2b illustrates the capacitance in the frequency range of 10 Hz to 10 MHz, validating the EDL effect of the MSQ electrolyte. The inset in Figure 2b shows the frequency-dependent capacitance curve characteristics of the EDL capacitor with a metal–insulator–metal (Al/MSQ electrolyte/Pt) structure. The capacitance at 10 Hz was approximately ~1.89 µF/cm^2^, and this value decreased as the frequency increased. This behavior could be explained by the frequency-dependent response of internal mobile protons. Lower frequencies provide a relaxed response time for internal mobile protons to accumulate at the interface [34]. In contrast, higher frequencies result in a more rapid proton response, leading to deteriorated capacitance [35]. Thus, the EDL effect of the proposed MSQ electrolyte could be verified by the frequency-dependent capacitance curve characteristics.

### 3.2. Electrical Characteristics of MSQ Electrolyte-Based EDL Synaptic Transistors

**Figure 3:**
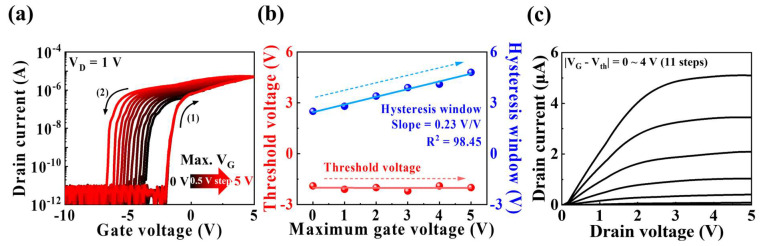
(**a**) Double-sweep transfer curves as a function of maximum gate voltage (*V*_G_ = 0–5 V in steps of 0.5 V) at constant *V*_D_ = 1 V. (**b**) Hysteresis window and threshold voltage with increase in maximum *V*_G_. (**c**) Output versus the difference in gate voltage and threshold voltage (|*V*_G_ − *V*_th_| = 0–4 V in steps of 0.4 V).

Figure 3a presents double-sweep transfer curves (*I*_D_–*V*_G_) of the fabricated MSQ-based EDL synaptic transistors. A constant drain voltage (*V*_D_ = 1 V) was applied, and the maximum gate voltage (*V*_G_) was increased from 0 V to 5 V in steps of 0.5 V. *V*_G_ underwent a forward sweep (1) followed by a backward sweep (2). The transfer curve characteristics exhibited counter-clockwise hysteresis, with the hysteresis window expanding with an increase in the maximum *V*_G_. The hysteresis phenomenon was attributable to slow polarization resulting from the movement of internal mobile protons in MSQ EDL. As the maximum *V*_G_ increased during the forward sweep, more mobile protons accumulated at the interface between the IGZO channel and MSQ electrolyte, increasing the number of charge carriers in the channel layer. Subsequently, protons diffused in the opposite direction during the backward sweep, increasing hysteresis in the counter-clockwise direction. Figure 3b shows changes in the hysteresis window and threshold voltage (*V*_th_) of the double-sweep transfer curve with increasing maximum *V*_G_. The hysteresis window increased with a slope of 0.23 V/V and linearity (R^2^) of 98.45, ranging from 2.5 to 4.8 V. Here, R^2^ serves as the linearity coefficient, evaluating how well the data within the hysteresis window fits the linear model. A linear trend is indicated by a linearity coefficient close to 1. Therefore, our measured hysteresis window has been demonstrated to increase linearly. Additionally, even as the maximum *V*_G_ increased, *V*_th_ remained unchanged at approximately −2 V. Figure 3c presents the output curve (*I*_D_–*V*_D_) measured with a 0.4 V step from 0 V to 4 V. As *V*_D_ increased, the drain current (*I*_D_) linearly increased, followed by pinch-off and saturation.

### 3.3. Synaptic Properties of Organic–Inorganic Hybrid MSQ-Based EDL Synaptic Transistors

**Figure 4:**
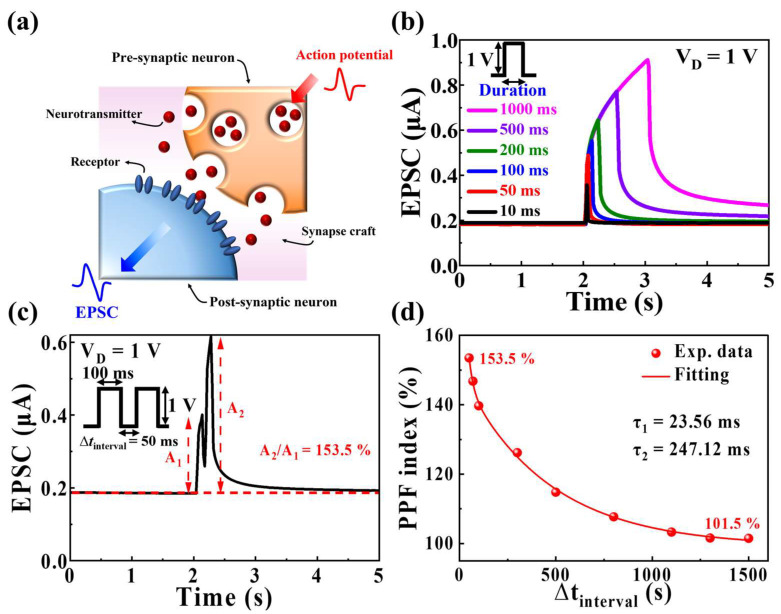
(**a**) Schematic of the operational principle of a biological synapse. (**b**) EPSCs for various durations (10–1000 ms) of a single pre-synaptic pulse with an amplitude of 1 V. (**c**) EPSCs demonstrating paired-pulse facilitation stimulated by a pre-synaptic paired-pulse spike (1 V, 100 ms) with ∆*t*_interval_ = 50 ms. (**d**) PPF index for various ∆*t*_interval_ (50 to 1500 ms) of the pre-synaptic spike.

Biological synapses serve as crucial structures for information transmission between neurons, occurring at the connection site between the axon and dendrite of nerve cells [14,36]. The pre-synaptic signal, conveyed from the axon, transforms into a neurotransmitter—a chemical substance released through the synaptic cleft [37]. This released neurotransmitter binds to receptors on the dendrite, transforming back into a post-synaptic signal, thereby generating EPSC [36,37]. Figure 4a schematically illustrates a biological synapse in the human brain. Understanding and emulating the mechanism and structure of biological synapses are critical for the successful implementation of synaptic transistors. In the organic–inorganic hybrid MSQ-based EDL synaptic transistors fabricated in this work, we designated the p-type Si bottom gate, IGZO channel, and MSQ-based EDL mobile proton as the pre-synapse, post-synapse, and neurotransmitter, respectively. Consequently, we measured the EPSC—the channel current generated by the electrical stimulation of the pre-synaptic gate—through the ITO (source/drain) electrode, emulating the operation of a biological synapse. Figure 4b presents the single-spike EPSC characteristic curve with a fixed spike amplitude of 1 V and pulse durations varying from 10 to 1000 ms at a constant *V*_D_ (=1 V). As the duration of the pre-synaptic pulse increased, the maximum EPSC and duration of EPSC retention after the spike also increased. This phenomenon could be explained as follows: with an extended spike duration, more mobile protons inside the MSQ diffused to the interface between the channel and MSQ electrolyte, increasing the conductivity and duration of EPSC after the spike [38,39]; therefore, the proposed organic–inorganic hybrid MSQ-based EDL synaptic transistor could modulate synaptic plasticity by altering the channel conductivity.

In the field of neuroscience, PPF is observed across various brain regions and neurotransmitter systems, representing a fundamental aspect of dynamic information processing and neuron communication [40,41]. PPF occurs when two consecutive stimuli are applied, resulting in the facilitation of the response to the second stimulus compared with the first. Figure 4c illustrates the EPSC triggered through the application of two identical pre-synaptic pulses (amplitude = 1 V; duration = 100 ms; ∆*t*_interval_ = 50 ms). The EPSC (*A*_2_) amplitude of the second spike was larger than the EPSC (*A*_1_) amplitude of the first spike. This behavior was attributable to the short interval between the first and second spikes, which caused the mobile protons in the MSQ to re-excite before complete relaxation following the first spike. Therefore, a narrow time interval (∆*t*_interval_) between spikes contributed to increased channel current, as the incomplete relaxation of the proton after the first spike led to proton generation by the second spike. The amplification ratio between the EPSCs following the first and second spikes was quantified using the PPF index. The PPF index was determined using the following double-exponential decay function: [42]

(1) $$PPF\;index={A+C}_{1}\exp(-\Delta t/\tau_{1})+C_{2}\exp(-\Delta t/\tau_{2}),$$

where *C*_1_ and *C*_2_ represent the initial values of the first and second spikes, respectively; and *τ*_1_ and *τ*_2_ denote the relaxation time after facilitation. For the fabricated synaptic transistor, *τ*_1_ and *τ*_2_ were 23.56 and 247.12 ms, respectively. Figure 4d presents the PPF index, indicating a high amplification of approximately 153.5% within a short time interval of 50 ms. The amplification decreased to approximately 101.5% at a longer time interval of 1500 ms. The following results demonstrated the effective modeling of the operational process of biological synaptic mechanisms.

**Figure 5:**
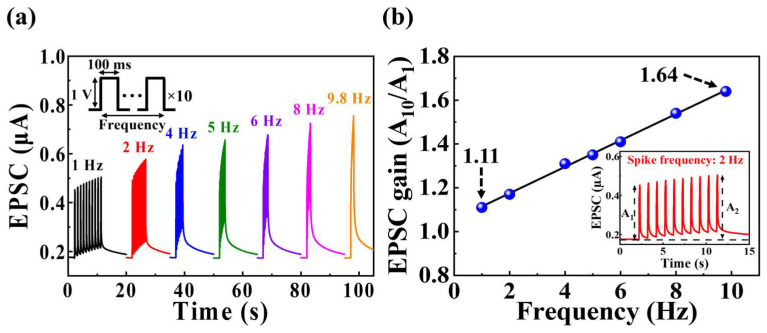
(**a**) EPSC responses to sequential pre-synaptic pulses (amplitude = 1 V; duration = 100 ms) with frequencies ranging from 1 to 10 Hz. (**b**) Variation in the EPSC gain (*A*_10_/*A*_1_) with the frequency of pre-synaptic spikes; the inset shows the EPSC response to 4 Hz.

Dynamic signal filtering based on frequency is facilitated by short-term synaptic plasticity. Short-term synaptic plasticity, primarily exhibited as short-term depression and facilitation, allows for variation in the response pattern of a neuron to repetitive stimuli, enabling signal pass filtering [43,44]. Therefore, the behavior of short-term synaptic plasticity, encompassing both depression and facilitation, contributes to low- and high-pass filtering functions [44]. Figure 5a illustrates the EPSC responses induced by 10 sequential pre-synaptic pulses (amplitude = 1 V; duration = 100 ms) with frequencies varying from 1 to 10 Hz. The EPSC values for each spike induced at a frequency of 1 Hz remained nearly constant, with minimal deviation from the first triggered EPSC value of ~0.45 µA. However, as the frequency increased, the tenth triggered EPSC increased. Figure 5b presents the frequency-dependent EPSC gain of pre-synaptic spikes, calculated as the ratio of the first EPSC gain (*A*_1_) to the tenth EPSC gain (*A*_10_). As the frequency increased from 1 Hz to 10 Hz, the EPSC gain increased from 1.11% to 1.64%. Thus, the proposed MSQ-based EDL synaptic transistor could successfully demonstrate high-pass filtering functionalities.

**Figure 6:**
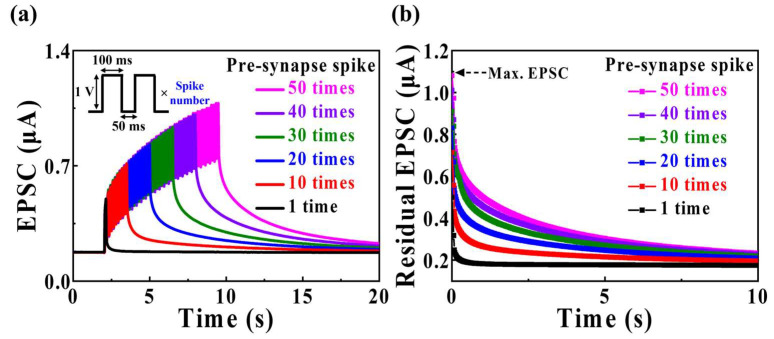
(**a**) EPSC response to pre-synaptic pulses (amplitude = 1 V; duration = 100 ms) with spike numbers varying from 1 to 50. (**b**) EPSC attenuation over time after pre-synaptic pulses for different numbers of spikes.

Synaptic plasticity is gradually enhanced with multiple consecutive pre-synaptic spikes. Figure 6a illustrates the EPSC responses to pre-synaptic pulses with spike numbers varying from 1 to 50 (amplitude = 1 V; duration = 100 ms). Applying multiple spike pulses to the gate led to a steady increase in the EPSC until the final spike stimulation. This observation suggests that as the number of spikes increases, the proton excited by the previous spike influences the proton excited by the next spike, resulting in a consecutive increase in EPSC values. Figure 6b depicts the decay of EPSC over time following the application of pulses for different numbers of spikes. In contrast to the EPSC from a single spike pulse, the EPSC from multiple spike pulses required a longer time to decay from the maximum value to the initial value. This behavior could be explained by the increase in the concentration gradient of mobile protons within MSQ with a higher number of pulses, extending the time to return to the initial equilibrium state. The increase in the maximum EPSC with the number of pre-synaptic spikes led to increased relaxation time, indicating a trend from short-term synaptic plasticity to long-term synaptic plasticity. Therefore, the fabricated MSQ-based EDL synaptic transistor demonstrated synaptic plasticity, attributable to the accumulation of mobile protons between the MSQ electrolyte and IGZO channel.

**Figure 7:**
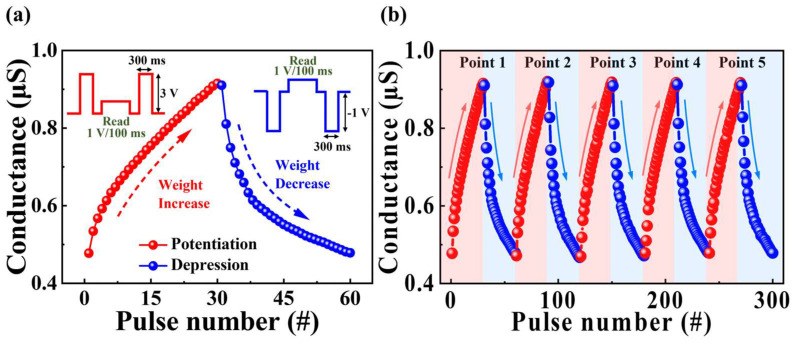
(**a**) Potentiation and depression characteristics of synaptic weights induced by pre-synapse pulses. (**b**) Endurance over five cycles of potentiation and depression.

Unlike short-term plasticity, which facilitates rapid information processing, long-term plasticity involves the adjustment of synaptic weights through mechanisms that either strengthen or weaken connections between neurons, facilitating the storage or erasure of information [45,46,47]. Long-term potentiation (LTP) and depression (LTD) occur in response to repetitive strong or weak stimuli, respectively. These processes play a crucial role in the learning and memory formation of neural networks by regulating synaptic weights between neurons in artificial synapses. By simulating LTP and LTD, the proposed synaptic transistor was anticipated to reliably replicate the mechanism of regulating synaptic strength observed in biological brains, thereby adjusting weights and contributing to the formation of learning networks. Figure 7a illustrates the modulation of synaptic weights in potentiation and depression through repetitive pre-synaptic stimuli, demonstrating the features of LTP and LTD. The single-cycle operation of potentiation and depression in pre-synaptic stimulation involved the application of enhancement and inhibition pulses to the gate electrode, each with amplitudes of 3 V and −1 V for 100 ms, respectively. A read pulse with an amplitude of 1 V and a duration of 300 ms was applied to measure changes in conductivity. The conductance increased (from 0.47 µA to 0.92 µA) and decreased (from 0.91 µA to 0.48 µA) owing to potentiation and depression operations, respectively. Figure 7b illustrates the continuous stability of conductivity during repetitive potentiation and depression over 5 cycles. Conductance modulation remained nearly constant throughout all cycles. Therefore, the proposed synaptic transistors have demonstrated the characteristics of LTP and LTD induced by repetitive stimulation. The following results demonstrated the effective modulation of synaptic weights through long-term repeated potentiation and depression. Thus, the fabricated organic–inorganic hybrid MSQ-based EDL synaptic transistor has successfully demonstrated the ability to mimic long-term information processing functions such as learning and memory in the brain by dynamically altering synaptic weights between neurons in neural networks.

### 3.4. MNIST DNN Simulation

**Figure 8:**
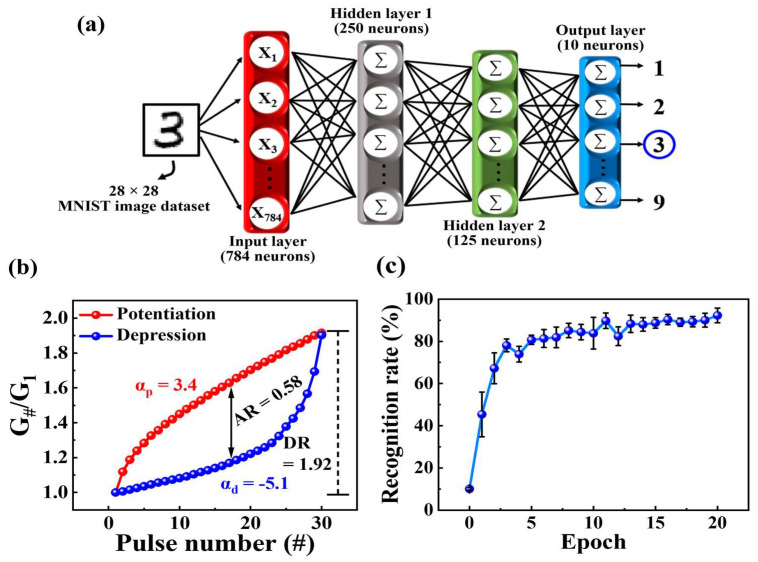
(**a**) Schematic of a DNN designed with input, hidden, and output layers, corresponding to the handwritten MNIST dataset consisting of 28 × 28 pixels. (**b**) Parametric analysis of normalized conductance for potentiation and depression. (**c**) Simulated recognition rates across varying numbers of epochs.

Artificial DNNs are machine learning algorithms composed of input, hidden, and output layers, designed to emulate the biological neuron network in the brain [48,49,50]. To demonstrate the neuromorphic computing capabilities of the proposed MSQ-based EDL synaptic transistors, we trained a multi-layer DNN using the IBM AIHWkit with the handwritten MNIST dataset [51]. Figure 8a illustrates the structure of the artificial DNN with an input layer (784 input neurons corresponding to the 28 × 28-pixel MNIST dataset), a hidden layer, and an output layer (10 output neurons associated with digits 0 to 9). The interlayer connections in this model were determined by synaptic weights, represented through normalized conductance (Figure 8b). The normalized conductance (*G*_#_/*G*_max_) was calculated by dividing each conductance (*G*_#_) by the maximum conductance (G_max_). Accuracy-related parameters, such as dynamic range (DR), asymmetric ratio (AR), and linearity characteristics, were derived through nonlinear analysis of normalized conductance. The DR, representing the modulated range of conductance, was calculated as the ratio of the maximum conductance to the minimum conductance, resulting in a value of 1.92. AR, reflecting the asymmetry in potentiation and depression, was calculated as [52]

(2) $$AR=\frac{\max\left|G_{p}\left(n\right)-G_{d}\left(n\right)\right|}{G_{p}\left(n_{max}\right)-G_{d}(n_{max})}\;\;for\;n=1\;to\;30,\;n_{max}=30,$$

where *G*_p_(*n*) and *G*_d_(*n*) represent the conductance for potentiation and depression at the *n*th pulse, respectively; and *n*_max_ is the maximum number of pulses for potentiation and depression. The AR for the proposed synaptic transistor was calculated to be 0.58, indicating enhanced symmetrical potentiation and depression, resulting in more accurate learning and recognition. To assess the linearity of updating synaptic weights, the nonlinearity parameter α was defined as [53]

(3) $$G=\left\{\begin{array}{c} {\left(\left(G_{max}^{\alpha }-G_{min}^{\alpha }\right)\times \omega +G_{min}^{\alpha }\right)}^{1/\alpha }\;,if\;\alpha \neq 0\;\\ G_{min}{\times (G_{max}/G_{min})}^{\omega }\;\;,if\;\alpha =o, \end{array}\right.$$

where *G*_max_ and *G*_min_ represent the maximum and minimum conductance, respectively; and ω is an internal variable ranging from 0 to 1. α represents the nonlinearity factor controlling potentiation and depression, with an ideal value of 1. The nonlinearity factors (α_p_ and α_d_) for potentiation and depression in the fabricated transistor were 3.4 and −5.1, respectively. Using the normalized conductance and extracted parameters, we designed the interlayer synaptic weights in the DNN model. The DNN was then trained over a dataset involving 60,000 MNIST images, and recognition simulation tests were performed using 10,000 handwritten images after each epoch of the training dataset. Figure 8c illustrates the recognition accuracy during training epochs, ranging from 0 to 20 cycles, with each node in the hidden layer set at constant values of 250 and 125. The recognition rate at epoch 0, representing the untrained state of the DNN model, was 10.1%, which increased to 92.28% as the number of epochs increased. These recognition simulation results demonstrated that the MSQ-based EDL synaptic transistor could successfully emulate human brain learning and memory processes.

## 4. Conclusions

This study represented the first attempt at establishing a synaptic transistor using organic–inorganic hybrid material MSQ as an electrolyte in the EDL. Through meticulous analyses involving frequency-dependent capacitance measurements, we validated the EDL characteristics, primarily governed by mobile protons within the MSQ electrolyte. The fabricated device exhibited remarkable electrochemical stability, as evidenced by a substantial capacitance of approximately 1.89 µF/cm^2^ at 10 Hz. Electrical evaluations involving double-sweep transfer curves revealed the occurrence of counterclockwise hysteresis, demonstrating the potential applications of the proposed device in neuromorphic computing. Moreover, the fabricated transistor displayed crucial biological synaptic transistor behaviors, encompassing short-term and long-term synaptic plasticity, along with signal high-pass filtering. Given its diverse synaptic characteristics, such as single-spike EPSC, PPF, frequency-dependent EPSC, spike-number-dependent EPSC, and potentiation/depression, the MSQ-based EDL synaptic transistor demonstrates significant potential for neuromorphic applications. Additionally, we extracted specific parameters from the normalized conductance and set the interlayer synaptic weights in the DNN model. Through repeated training over handwritten MNIST datasets, the DNN achieved an outstanding recognition rate of 92.28%. Overall, the proposed polymer organic–inorganic hybrid MSQ-based EDL synaptic transistor, characterized by excellent electrical stability, represents a promising high-performance next-generation artificial synaptic device with enhanced synaptic functionality and compatibility with CMOS due to improved thermal and chemical stability.

## Data Availability

Data are contained within the article.
